# The American Institute of Homeopathy

**Published:** 1897-05

**Authors:** Dewitt G. Wilcox


### THE AMERICAN INSTITUTE OF HOMEOPATHY.

The next meeting of the American Institute of Homeopathy will be held in Buffalo, June 23d to 30th, 1897. The local committee wishes to see every homeopathic physician of the United States present at this meeting. The surest way to come is to make your plans early, and be determined to carry them out.

One other thing we wish to do is to get every homeopathic physician who is not a member of the Institute to join at this coming meeting. There is no better time or opportunity to join than now. Kindly fill out a blank and forward it, together with the necessary fee, to the undersigned. We will see that the application is properly indorsed if you are eligible to membership.

If you already belong to this body will you kindly lend your services in securing a new member for the American Institute. Address

DEWITT G. WILCOX,
173 Lexington Avenue.

Chairman Sub-Committee on Invitations and New Members of American Institute.

